# Hydrolytic secretome engineering in *Yarrowia lipolytica* for consolidated bioprocessing on polysaccharide resources: review on starch, cellulose, xylan, and inulin

**DOI:** 10.1007/s00253-021-11097-1

**Published:** 2021-01-15

**Authors:** Ewelina Celińska, Jean-Marc Nicaud, Wojciech Białas

**Affiliations:** 1 Department of Biotechnology and Food Microbiology, Poznan University of Life Sciences, ul. Wojska Polskiego 48, 60-627 Poznań, Poland; 2 Micalis Institute, INRAE-AgroParisTech, UMR1319, Team BIMLip: Integrative Metabolism of Microbial Lipids, Domaine de Vilvert, 78352 Jouy-en-Josas, France

**Keywords:** Complex substrates, Residual biomass, Genetic engineering, Yeast, Heterologous gene, Polymer hydrolysis

## Abstract

**Abstract:**

Consolidated bioprocessing (CBP) featuring concomitant hydrolysis of renewable substrates and microbial conversion into value-added biomolecules is considered to bring substantial benefits to the overall process efficiency. The biggest challenge in developing an economically feasible CBP process is identification of bifunctional biocatalyst merging the ability to utilize the substrate and convert it to value-added product with high efficiency. *Yarrowia lipolytica* is known for its exceptional performance in hydrophobic substrates assimilation and storage. On the other hand, its capacity to grow on plant-derived biomass is strongly limited. Still, its high potential to simultaneously overproduce several secretory proteins makes *Y. lipolytica* a platform of choice for expanding its substrate range to complex polysaccharides by engineering its hydrolytic secretome. This review provides an overview of different genetic engineering strategies advancing development of *Y. lipolytica* strains able to grow on the following four complex polysaccharides: starch, cellulose, xylan, and inulin. Much attention has been paid to genome mining studies uncovering native potential of this species to assimilate untypical sugars, as in many cases it turns out that dormant pathways are present in *Y. lipolytica*’s genome. In addition, the magnitude of the economic gain by CBP processing is here discussed and supported with adequate calculations based on simulated process models.

**Key points:**

*• The mini-review updates the knowledge on polysaccharide-utilizing Yarrowia lipolytica.*

*• Insight into molecular bases founding new biochemical qualities is provided.*

*• Model industrial processes were simulated and the associated costs were calculated.*

**Supplementary Information:**

The online version contains supplementary material available at 10.1007/s00253-021-11097-1.

## Introduction

**Fig. 1:**
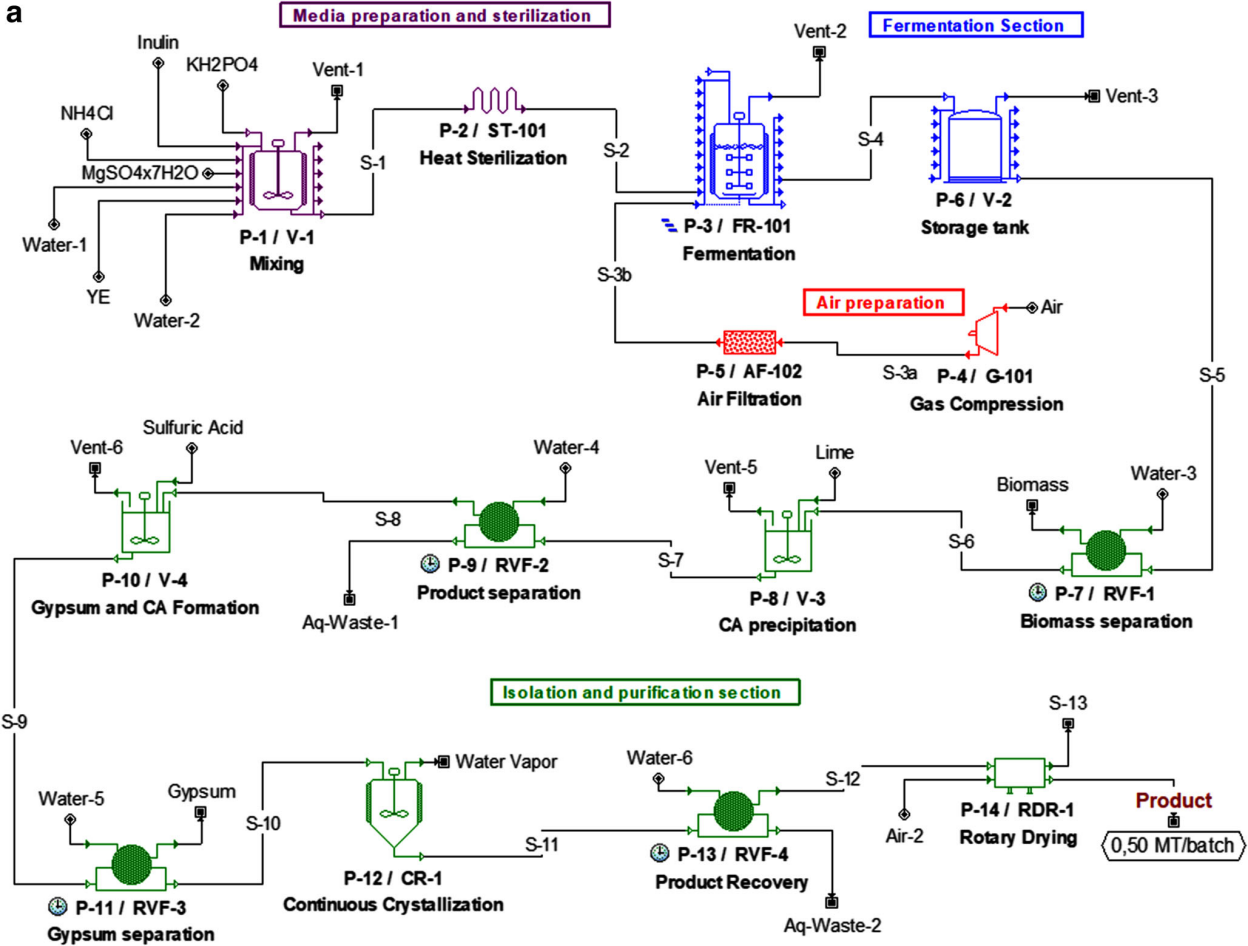

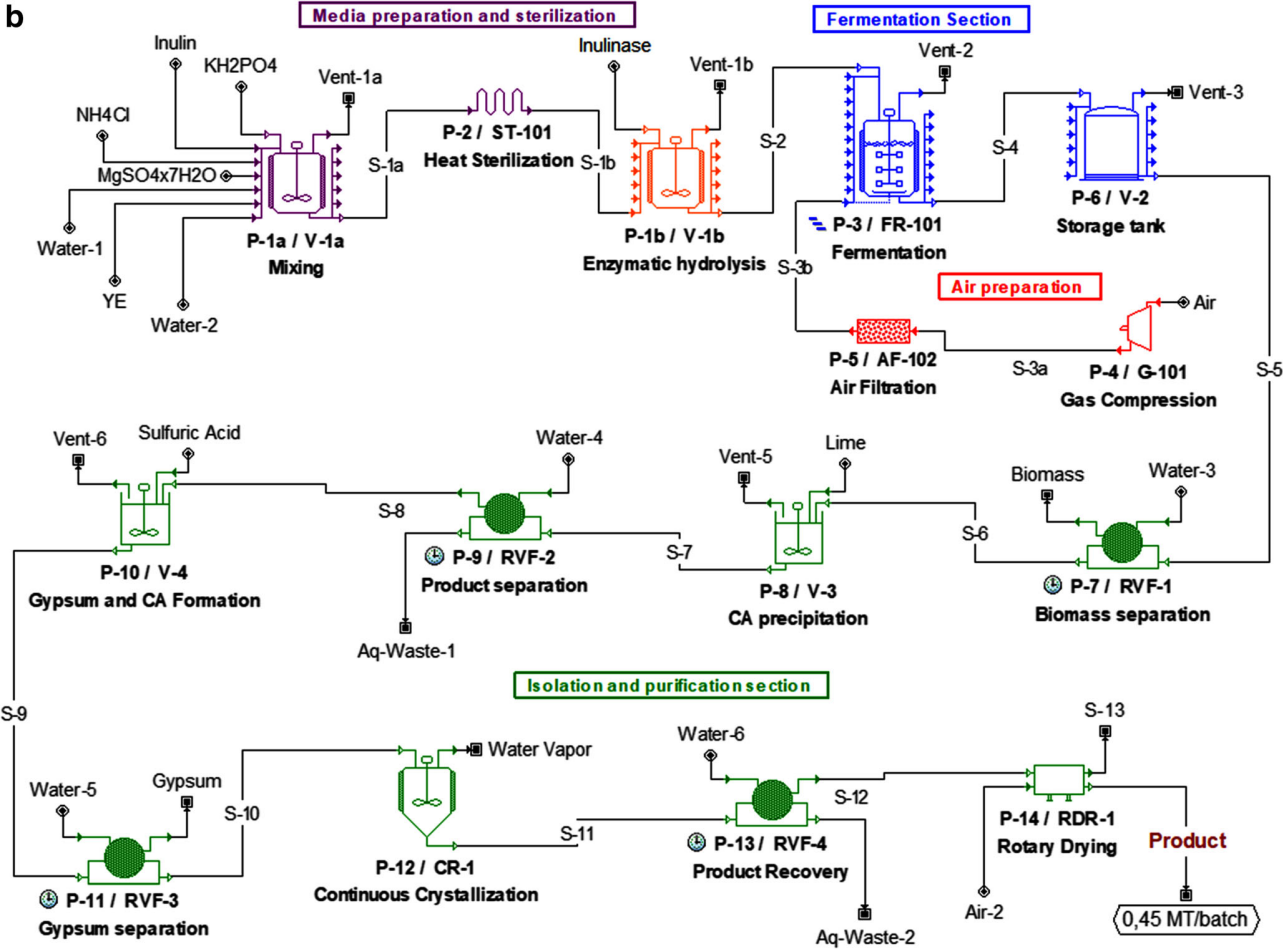
Schematic representation of a model process executed via **a** CBP concept vs. **b** enzymatic-cocktail-based process of inulin conversion into citric acid by *Y. lipolytica*. The flowsheet of the model processes and all the calculations were prepared with SuperPro Designer software. CA—citric acid, P—process, V—vessel, S—stream, ST—sterilizer, FR—fermenter, AF—air filtration unit, G—gas compressor, RVF—rotary vacuum filter, CR—crystallizer, RDR—rotary dryer, clock icon—equipment operating in cycles, which can be exploited continuously. Blue dashes indicate several bioreactors operating in time shifts for better exploitation of the production line

Consolidated bioprocessing (CBP) featuring concomitant production of hydrolases active toward polymeric substrates and microbial conversion of the released consumable sugars into value-added biomolecules in a single step holds promise for cost-effective complex biomass conversion. The key prerequisite in realization of the CBP concept is identifying a biocatalyst merging the two critical traits: (1) to decompose complex feedstock at high rates and (2) to produce desired compounds in a commercially relevant manner. As the nature favors survival rather than high-level production and abundance, one or both of the traits have to be somehow engineered, by bioprocessing and/or genetic engineering approaches. Typical industrial processes relying on complex polysaccharides conversion support the former critical trait by implementation of enzymatic cocktails facilitating rapid and complete substrate decomposition. However, the cost of such enzymatic preparation may account for approximately 30% of the capital costs (Sánchez and Cardona 2008). To support this widely accepted and frequently quoted statement with adequate numbers, we have prepared two variants of a simulated process model and calculated associated costs for (A) a process conducted according to the CBP concept, and (B) a process implementing the raw material treatment with an enzymatic cocktail (Fig. 1; Tables S1–S4). The process models use a renewable polysaccharide—inulin—and citric acid as the target molecule. All the presumptions were based on literature data on inulin-based bioprocesses, citric acid synthesis from inulin (Han et al. 2017; Singh et al. 2018; Rakicka et al. 2019), and market search for industrial materials prices (MP). In comparison to the simulated CBP process, the cocktail-based process encompasses additional procedure P1b (Fig. 1) conducted at elevated temperature (50 °C) over 48 h which is required for inulin hydrolysis. The P1b process reaches maximally 90% of conversion (Singh et al. 2018), which contributes to a proportional reduction in batch throughput by 10% in kilograms MP/batch. But foremost, the CBP process, due to elimination of the hydrolysis (P1b) procedure, is significantly shorter (batch time is reduced by 1.63-fold; from ~ 10 to ~ 6 days), thus enabling execution of 410 batches per year (vs. 148 batches annually for a traditional process with a separate hydrolysis procedure). Lowered yield plus longer time per a single batch renders the annual throughput of the traditional process over 3-fold lower in kilograms/year than the CBP. Accordingly, the additional P1b procedure renders the traditional process more demanding in terms of required power input by nearly 15% in kilowatt-hours/batch. Finally, the cost of commercial enzymatic cocktail enabling inulin decomposition into assimilable sugars constitutes > 21% of total material cost in the traditional process (Tables S3–S4). Together with the remaining material constituents, the unit production cost of the traditional process is by > 40% higher than the CBP (8.49 vs. 6.03 in $/kg MP) (Tables S3–S4).

Therefore, in order to relieve the complex polysaccharide-based bioprocesses from the economic burden of the enzyme purchase/production, efforts toward establishing consolidated biocatalysts are being pursued. Providing that the second critical trait of a biocatalyst is fulfilled, the main challenge in establishing consolidated biocatalyst is developing a strain with highly active hydrolytic secretome, encompassing all the enzymatic activities required for decomposition of the complex substrate. To this end, microorganisms with highly efficient translational-secretory machinery are of high interest and importance.

*Yarrowia lipolytica* is a non-conventional yeast species of high industrial relevance (Groenewald et al. 2014). It is mainly known for its high capacity to assimilate hydrophobic substrates and to produce an array of value-added products, like organic acids (Rywińska and Rymowicz 2011; Rywinska et al. 2012), erythritol (Mirończuk et al. 2016; Rakicka-Pustułka et al. 2020), aromas (Celińska et al. 2013; Celińska et al. 2019), and microbial lipids (Papanikolaou and Aggelis 2002; Beopoulos et al. 2008; Beopoulos et al. 2009). While *Y. lipolytica* was proved to grow very efficiently on a variety of different waste substrates, like crude glycerol (Papanikolaou and Aggelis 2002; Rakicka et al. 2015; Dobrowolski et al. 2016; Gajdoš et al. 2017), industrial wastes of tallow (Papanikolaou et al. 2007), or olive-mill waste water (Papanikolaou et al. 2008), its capacity to decompose renewable plant biomass is strongly limited (Barth and Gaillardin 1996; Kurtzman and Fell 2006). The innate ability of this species to assimilate monomeric products of the polysaccharides decomposition is also restricted, as the pathways for cellobiose, sucrose, maltose, xylose, arabinose, and galactose assimilation are either cryptic or absent from *Y. lipolytica*’s genome. On the other hand, *Y. lipolytica* is known for its exceptional capacity for overproduction of secretory proteins (Theron et al. 2020) owing to its unusual secretory pathway (Celińska and Nicaud 2018). Such a trait makes *Y. lipolytica* a platform of choice for expanding its substrate range to complex polysaccharides by engineering its hydrolytic secretome. Indeed, significant efforts have been made to endow *Y. lipolytica* strains with the ability to utilize complex polysaccharides, derived from renewable biomass.

The present paper provides an overview of different genetic engineering strategies advancing development of *Y. lipolytica* strains able to grow on the following four complex polysaccharides: starch, cellulose, xylan, and inulin. Much attention has been paid to investigations into native potential of this species to assimilate products of such hydrolysis, as in many cases it turns out that dormant pathways of untypical sugars assimilations are present in *Y. lipolytica* genome. This review updates the previous comprehensive article by Ledesma-Amaro and Nicaud (2016) and expands the scope of another excellent, recent paper (Spagnuolo et al. 2018) by focusing on genetic engineering of hydrolytic secretome for development of a consolidated biocatalyst strain.

### Starch

Starch is one of the most abundant polysaccharides in nature, composed of glucose monomers joined with alpha-glycosidic bonds (α-(1,4), branch-points at α-(1,6)). For industrial applications, it is extracted from various agricultural raw materials, including corn, potato, cassava, wheat, and other sources. Depending on its biological origin, it may contain different relative amounts of two isoforms—linear amylose and branched amylopectin. In its native or processed form, it is widely used in food and feed production, textile, pharmaceutical and paper industries or, more recently, for renewable biopolymer production. While gradually replaced by lignocellulosic biomass (which is not competing with the food sector), starch is still the most widely utilized substrate for biofuel production. In addition, starch-rich food waste and by-product streams generated by bakery, confectionery, and wheat-milling plants emerge as a potential feedstock for the synthesis of microbial bioproducts (Tsakona et al. 2014; Tsakona et al. 2019).

**Fig. 2:**
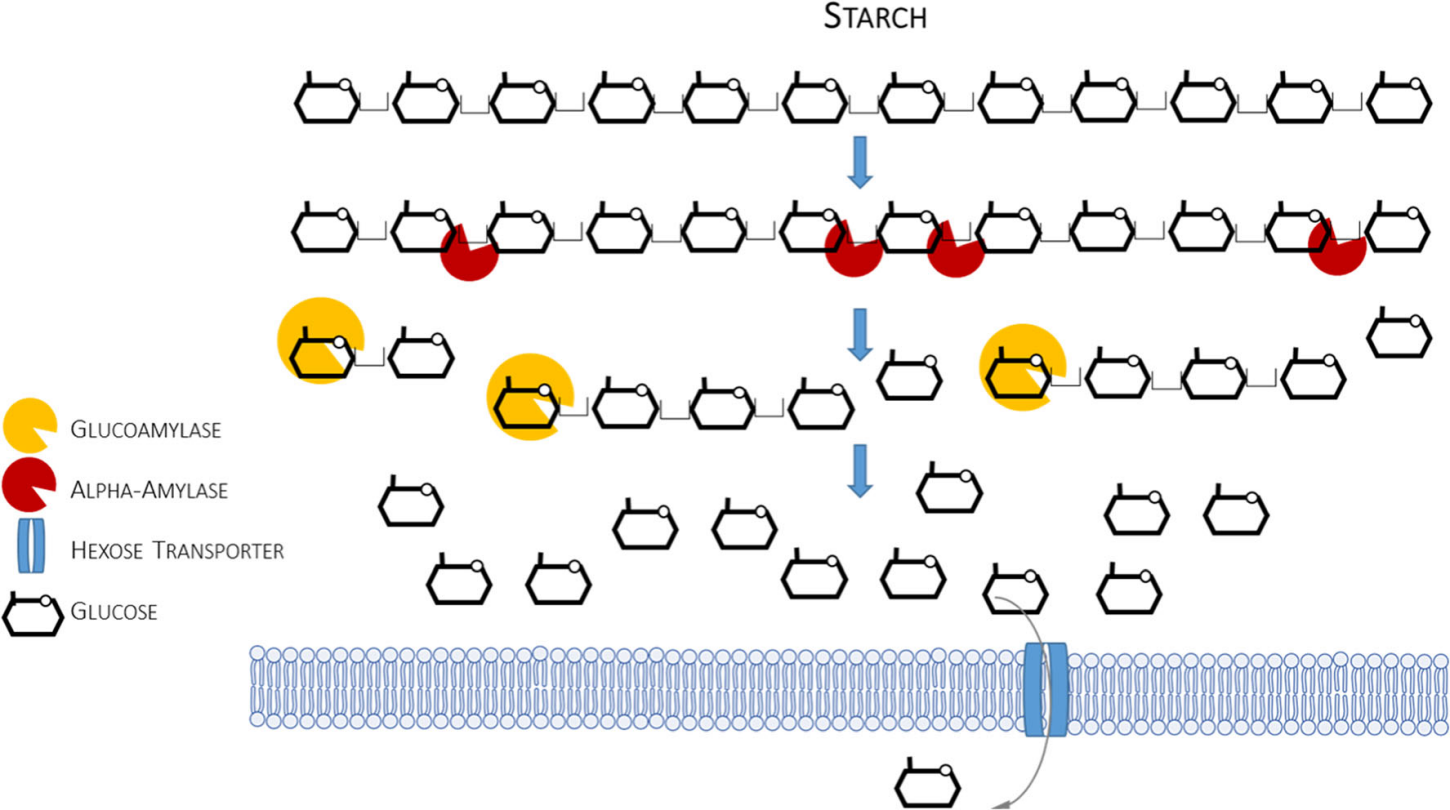
Simplified scheme of enzymatic decomposition of starch. Only those enzymatic activities that were engineered in *Y. lipolytica* (or the elements studied in *Y. lipolytica*, like protein transporters) are depicted in this simplified scheme

While a variety of different amylolytic enzymes are distinguished based on specificity of action (including pullulanase, alpha-amylase, and isoamylase), practically only two enzymatic activities are required for nearly complete decomposition of starch to simple sugars that are sufficient to support growth of microorganisms. These are alpha-amylase, which is an endoglucanase that catalyzes random hydrolysis of endo α-(1,4) glycosidic bonds in starch, and glucoamylase, which cleaves α-(1,4) glycosidic bond at the non-reducing end of starch, as well as α-(1,6) glycosidic bonds at branch points of amylopectin (Fig. 2). The origin of the majority of market enzymatic preparations are fungi (in particular *Aspergillus* spp.) and specific bacilli, for example, *Bacillus amyloliquefaciens*. None of such amylases has been identified in *Y. lipolytica* to date, and wild-type strains cannot degrade starch polymer supplied in the culture medium.

Like the other fungi, *Y. lipolytica* is able to accumulate and degrade intracellularly stored glycogen, which has the same structure as amylopectin (the branched isoform of starch). Glycogen synthesis in *Y. lipolytica* is executed by a single non-essential gene (YALI0F18502g; *GSY1*) encoding glycogen synthase (Bhutada et al. 2017). Mobilization of the storage material takes place upon depletion of nutrients and under stress conditions, as exemplified by nitrogen limitation conditions (Bhutada et al. 2017). The genes responsible for glycogen degradation were not systematically studied in *Y. lipolytica*; however, identity search (blastp) of glycogen phosphorylase (*GPH1*) and glucoamylase/glucan 1,4-α-glucosidase (*SGA1*) from *Saccharomyces cerevisiae* against *Y. lipolytica* proteome renders a single glycogen phosphorylase (YALI0F04169p) and a single six-hairpin glycosidase-like protein (YALI0E05203p) with positives of 75% and 48%. Nonetheless, amylolytic phenotype, understood as the ability to decompose “starchy polymer” provided in the culture medium into consumable sugars, was not reported for wild-type *Y. lipolytica*. On the other hand, the literature review showed that starch-decomposing enzymes were among the first heterologous genes cloned in *Y. lipolytica* host, e.g., α-amylase from rice (*Oryza sativa*) (Park et al. 1997) or thermostable α-amylase from *Thermobifida fusca* (Yang et al. 2010). At first, the purpose of the amylases cloning was to study heterologous protein synthesis in *Y. lipolytica* and to use the amylolytic activity as an easy-to-follow enzymatic reporter (Park et al. 1997; Dulermo et al. 2017). The former report on the plant α-amylase was followed by a series of papers on advancing production of the heterologous protein in *Y. lipolytica* by adopting different bioprocessing solutions (cyclic fed-batch, high cell density etc.), rather than to endow the yeast with efficient starch-decomposing phenotype (Chang et al. 1998a, b; Kim et al. 2000). Nevertheless, the information gained from that research facilitated usage of the rice α-amylase in the following studies where *Y. lipolytica* was actually transformed into a consolidated biocatalyst growing on raw starch (Ledesma-Amaro et al. 2015). By combination of the rice α-amylase with glucoamylase from *Aspergillus niger*, the authors constructed the first *Y. lipolytica* strain able to grow on starch. Both genes were expressed under control of a strong, constitutive TEF promoter, and their native signal peptides were replaced by a pre-signal sequence of the main extracellular lipase, Lip2p, followed by three X-Ala motifs. Systematical comparison of different signal peptides, including Lip2p and Lip2p-3-X-Ala, proved superiority of the latter (Celińska et al. 2018). The added value of that engineering approach was that the hydrolytic secretome of the constructed strain was active toward native, non-pretreated starch, which is of great industrial importance. The substrates used in that study were wheat starch and industrial product containing starch (DZ starch) provided by Tereos Syral (Belgium). In addition, when the “amylolytic phenotype expression cassette” was transformed into “obese” strain background (*Y. lipolytica* modified for enhanced accumulation of lipids (Beopoulos et al. 2008; Beopoulos et al. 2012)), the strain accumulated 27% of DCW as fatty acids directly from raw starch.

A second series of studies on starch-digesting *Y. lipolytica* differed from the previous one by the key biocatalyst α-amylase, which initiates the process of starch decomposition and thus it dictates its paste. In this second series, an insect gene from a rice pest (*Sitophilus oryzae*) was cloned and expressed in *Y. lipolytica*. The initial studies on feasibility of the insect gene’s expression in *Y. lipolytica* (Celińska et al. 2015) were followed by optimization of the enzyme synthesis in different genetic background (Celińska et al. 2016a), manipulation with signal peptides for improved secretion (Celińska et al. 2018), developing fast and reliable screening methods for rapid evaluation of numerous strain variants (Borkowska et al. 2019; Soudier et al. 2019), and to finally test different bioprocessing solutions to improve heterologous production of the protein (Celińska et al. 2017). Properties of the heterologous α-amylase were analyzed to verify its raw starch digesting potential (Celińska et al. 2016b) and compare it with commercial preparations. The amylase was proved to act on rice, amaranth, and pea starches in raw state and a broad panel of liquefied starches of various plant origin. The knowledge gained from those studies was applied in the following rationale-driven optimization of *Y. lipolytica*–based consolidated biocatalyst through combinatorial cloning of different signal peptides and changing positional order of the α-amylase and glucoamylase genes in double gene expression cassettes (Celińska et al. 2020). The aforementioned insect α-amylase was cloned in tandem with *Thermomyces lanuginosus* glucoamylase having industrially relevant characteristics (Favaro et al. 2015). Feasibility of the *T. lanuginosus* glucoamylase gene’s expression and secretion was tested in advance (unpublished). As for the α-amylase, the glucoamylase was also transcriptionally fused with ten different signal peptides and efficiency of the enzyme secretion was evaluated (Celińska et al. 2018). Combined expression of the two genes endowed *Y. lipolytica* with amylolytic phenotype, active on rice, potato, and corn starches in liquefied and native form. Depending on the signal peptide, the order of genes within the expression cassette, and the type of substrate, the growth of the obtained recombinant strain differed significantly. The level of microbial lipid accumulation from raw starch in the best consolidated biocatalyst strain (the amylases fused to a signal peptide of YALI0B03564p; glucoamylase in the first position and α-amylase in the second position of the expression cassette) was comparable with the typical values obtained with wild-type *Y. lipolytica* strains cultured on assimilable substrates, like glycerol or glucose, suggesting that carbon provision was not the limiting factor. In addition, the optimized strain was further used as a producer of a raw starch digesting enzymatic preparation composed of the two enzymatic activities (Gęsicka et al. 2020). The production was conducted in fed-batch bioreactor cultures on glycerol. A partly purified preparation obtained from *Y. lipolytica* post-culturing medium was proved operable in biotechnological production of ethanol and lactic acid conducted according to simultaneous saccharification and fermentation concept.

### Cellulose

Cellulose is the main constituent of lignocellulosic biomass, being a basic structural component of plants’ cell wall. Cellulose is mainly provided to the market by processing of wood pulp and cotton. The vast majority of marketed cellulose is used in papermaking and in food industry. In contrast to starch, its usage in biotechnology does not raise concerns regarding competitions with the food sector. Due to its great abundance, availability, and low price, it is considered a highly attractive substrate for biotechnological transformations.

**Fig. 3:**
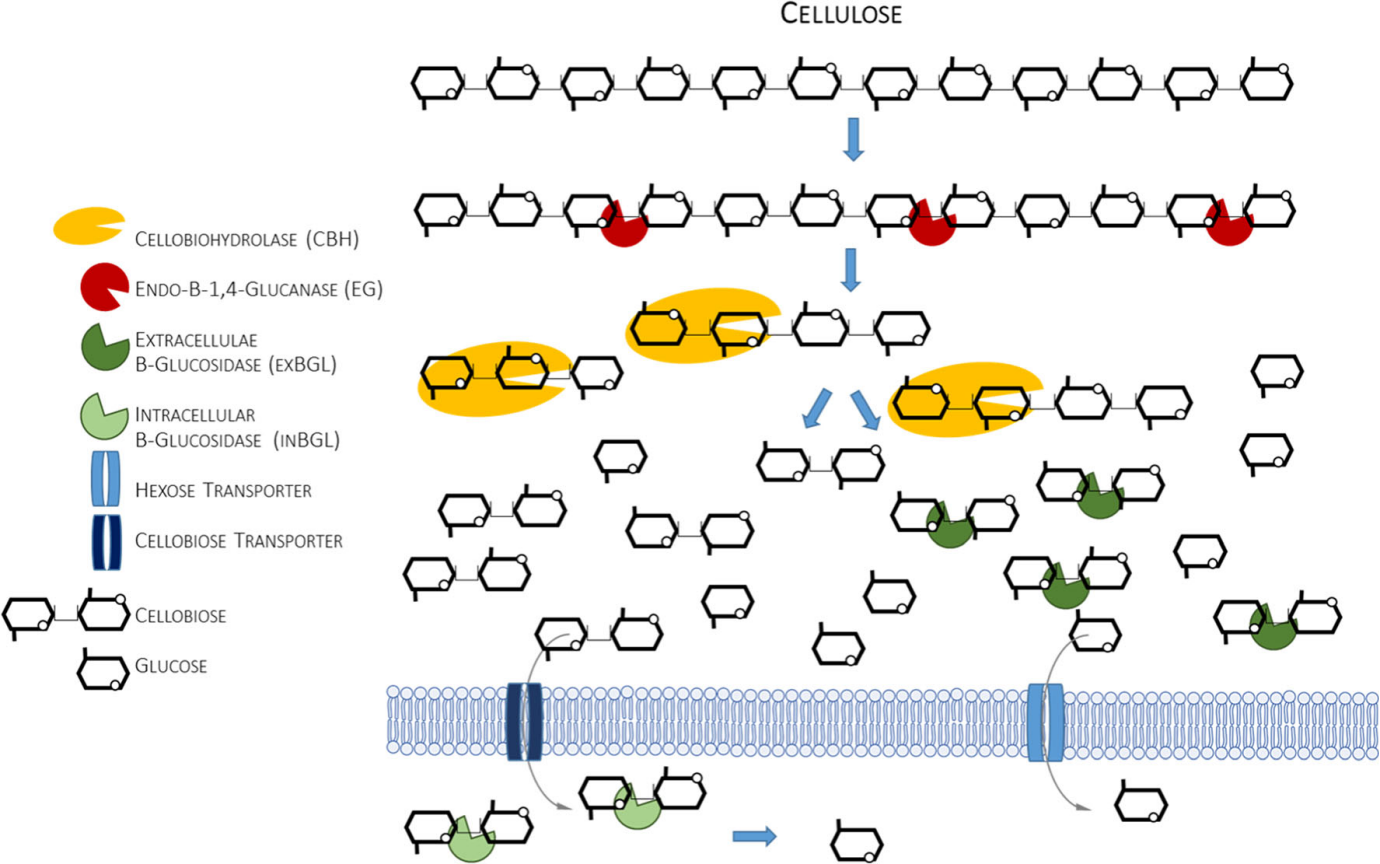
Simplified scheme of enzymatic decomposition of cellulose. Only those enzymatic activities that were engineered in *Y. lipolytica* (or the elements studied in *Y. lipolytica*, like protein transporters) are depicted in this simplified scheme

In raw lignocellulosic substrate, cellulose is accompanied by hemicelluloses and lignins that tightly surround cellulosic microfibrils, which typically account for ~ 60% of its dry matter content. Cellulose is homogenous in terms of chemical composition, as it is built solely of β-1,4-linked glucose residues, but may exist in either amorphous or highly ordered crystalline form. Condensed structure and complexity of lignocellulosic material makes it recalcitrant to enzymatic treatment in its raw state. Thus, prior to conversion of the cellulosic fraction into fermentable sugars, the raw, complex biomass must be first pretreated to break down the lignocellulose matrix and release the cellulosic microfibrils. Once these are obtained, a repertoire of enzymatic activities are required to decompose the polymer into monomers. The minimal enzymatic set comprises endoglucanases (EGs; EGI and EGII), cleaving internal bonds in the β-glucan chain, and cellobiohydrolases (CBHs) that act at the polymer extremities and release cellodextrins, i.e., cellobiose (dp2), which are then converted into monomeric glucose by the action of β-glucosidases (BGLs) (Fig. 3). The vast majority of cellulolytic preparations are composed of *Trichoderma reesei* cellulolytic secretome’s elements. Detailed studies conducted on this set of enzymatic proteins revealed several key facts that were later on applied upon engineering of the cellulose-degrading secretome in *Y. lipolytica*. In was for example revealed that both EG activities (EGI and EGII) play important and complementary roles in cellulose degradation, and they cannot be used interchangeably. In the following studies, it was revealed that EGI is more active toward the most recalcitrant fractions of cellulose, exhibiting twice the activity of EGII (Guo et al. 2017a). CBHs are also represented by two activities (Cel7A and Cel6A in *T. reesei*) being the major extracellular cellulases, representing 50% and 20% of the total amount of the protein, respectively (w/w) (Park et al. 2000).

Interestingly, *Y. lipolytica*’s genome bears several elements of a dormant cellulose, or, more precisely, cellobiose degradation pathway (Guo et al. 2015; Ryu et al. 2016). Literature reported some examples of isolation of cellobiose-grown *Y. lipolytica* strains (Kurtzman and Fell 2006). In addition, laboratory strains can be adapted to cellobiose (and xylose) utilization via repeated subculturing in these substrates (Ryu et al. 2016). *Y. lipolytica* is not able to grow on polymeric cellulose, as only terminal elements of the cellulose degradation cascade are present in the genome, without the elements of hydrolytic secretome. Regarding the cellobiose assimilation and degradation, it can follow one of two possible routes (Fig. 3): (1) extracellular BGLs hydrolyze cellobiose into two glucose moieties which are imported into the cell *via* hexose transporters, (2) cellobiose is directly transported into the cell via specific cellobiose transporter, and intracellular BGLs convert it to glucose. Genome mining analyses conducted by Guo et al. (2015) predicted six BGLs in *Y. lipolytica*’s genome, three intracellular (YALI0F16027g, YALI0D18381g, YALI0B14289g) and three extracellular (YALI0B14333g, YALI0E20185g, YALI0F01672g). The following work (Ryu et al. 2016) predicted five additional BGLs, YALI0B03564p, YALI0E33539p, YALI0E21109p, YALI0F01947p, and YALI0F13299p, which were transcriptionally responsive in the presence of cellobiose. Insightful analysis at the transcriptional level demonstrated that in the presence of cellobiose, only three intracellular BGL genes were upregulated—cytosolic YALI0E20185g, YALI0F01672g, and nuclear YALI0F13299g—while none of the extracellular BGLs nor three remaining intracellular BGLs were responsive to those conditions. Those results could suggest that in the wild-type *Y. lipolytica*, cellobiose is assimilated solely intracellularly, and a specific cellobiose transporter is involved in this process. According to analysis conducted by Ryu et al. (2016), four putative cellobiose transporters are encoded in *Y. lipolytica*’s genome: YALI0D01111g, YALI0C04730g, YALI0D00363g, and YALI0B00396g. Functional overexpression of YALI0D01111g endowed the recombinant strains with the ability to completely assimilate cellobiose and outperform the parental strain, which assimilated the disaccharide with low efficiency. Nevertheless, further analyses conducted by those authors indicated that when *Y. lipolytica* was grown in a mixture of cellobiose and xylose, most of the extracellular and intracellular BGLs were induced, suggesting that native *Y. lipolytica* strains could degrade cellobiose both intracellularly and extracellularly (Ryu et al. 2016).

In terms of hydrolytic secretome engineering toward utilization of cellulose/cellobiose by *Y. lipolytica*, the first studies concerned cloning of individual genes, i.e., cellulases from *Aspergillus aculeatus* and *Humicola insolens* (Muller et al. 1998). However, as in the case of initial works on the amylolytic *Y. lipolytica* strains, the enzymes were cloned as the reporting agents. The following work concerned high-level overproduction of *T. reesei* EGI (TrEGI; Tr is for *T. reesei*), which was an exemplification of *Y. lipolytica*’s potential as an expression platform (Park et al. 2000). Comparative cloning of *T. reesei* genes encoding EGII and CBHII was conducted to quantitatively describe the level of the gene overexpression from different DNA constructions and in different yeast hosts (Boonvitthya et al. 2013). Likewise, the problem of low production of TrCBHI was addressed by generating a fusion protein, led by easily processed TrEGII, and its expression in three yeast expression platforms—*S. cerevisiae, Y. lipolytica*, and *Lipomyces starkeyi* (Xu et al. 2018). While the fusion protein approach alleviated low synthesis of CBHI in *L. starkeyi*, in *Y. lipolytica* the TrCBHI (in fact a chimeric protein *T. reesei–Talaromyces emersonii* TrTeCBHI) was produced at higher amounts without the fusion partner. Interestingly, digestion of pretreated corn stover with the engineered secretomes of *Y. lipolytica* and *L. starkeyi* showed that conversion was much better using *Y. lipolytica* secretome (50% vs. 29%, respectively).

The chimeric TrTeCBHI was used earlier in a study on individual cloning of *T. reesei* EGII, CBHII, and the TrTeCBHI in *Y. lipolytica* (Wei et al. 2014). The concept was to co-culture the three strains expressing complementary activities, which indeed enabled consumption of cellulose contained in the medium, however at moderate level (23%). Nevertheless, the authors were the first to report on incompatibility of *T. reesei*’s CBHI with *Y. lipolytica* expression system, which, as noted there, can be expressed in a native form, but its activity remains below expectation. Therefore, prior to selection of the strains/activities to the final co-culture, the authors tested the other CBHI genes—apart from native CBHI from *T. reesei*, the CBHI from *Chaetomium thermophilum, Humicola grisea*, and the aforementioned chimeric protein TrTeCBHI. As the chimera performed the best toward recalcitrant cellulose (Avicell) and showed significant synergism with EGII and CBHII in degrading cellulosic substrates, it was chosen for the final tests with mixed supernatants and the three-strain co-culture. The chimeric TrTeCBHI was also co-cloned under a strong constitutive promoter with the remaining cellulose-degrading activities, EGII and CBHII from *T. reesei*, as a single integrative expression cassette (Wei et al. 2019). The DNA construction was cloned in a “lipidogenic” strain’s background resulting in observable conversion of consumed cellulose to lipids. Noteworthy, the authors observed that high-level overexpression of heterologous secretory proteins caused a drain in the ER (endoplasmic reticulum; site of folding, maturation, and initiation of polypeptide secretion), leading to competition between the cellulase formation and the lipid synthesis, which is also initiated in the ER. It was then pointed that the intrinsic link between cellulase co-expression/secretion and lipid accumulation may hamper generation of high-level lipid production from cellulose by recombinant *Y. lipolytica* (Wei et al. 2019).

With the aim to boost the potential of *Y. lipolytica* to utilize cellobiose, heterologous counterparts of a cellobiose transporter (Nc_cdt-1) and the BGL activity (Nc_gh1_1; Nc—*N. crassa*) from *Neurospora crassa* were overexpressed in *Y. lipolytica* under a strong hybrid promoter (Lane et al. 2015). Apart from generating cellobiose-consuming strain, the authors observed that *Y. lipolytica* can consume cellobiose in the presence of glucose (so it is not subjected to carbon catabolite repression in this regard), and, as it was also investigated and observed by Ryu et al. (2016), that cellobiose transportation is the limiting step in the cellobiose assimilation pathway. Native BGL gene activation was also recently conducted using an innovative synthetic biology tool, namely CRISPRa (CRISPR-dCas9 activation) (Schwartz et al. 2018). The authors constructed and validated a CRISPR-based genome editing tool using a synthetic gene expression activator (VPR) and used it for activation of the dormant BGL expression in *Y. lipolytica*, generating a strain with enhanced growth on cellobiose.

Undoubtedly, the most comprehensive quest toward cellulolytic *Y. lipolytica* was described by a French group in a series of articles (Guo et al. 2015, 2017a, b, 2018). The first paper out of this series aimed at awaking *Y. lipolytica*’s native cellulolytic pathway, by overexpressing the six identified BGLs (mentioned above). As the authors observed, only the strains overexpressing two BGLs, YALI0F16027g (BGL1; intracellular membrane bound) and YALI0B14289g (BGL2; secreted), were able to degrade cellobiose, while the other four did not display any detectable activity. The strain overexpressing the two BGLs was further used as the background for cloning of additional cellulolytic activities, i.e., TrEGI, TrEGII, NcCBHI, and TrCBHII (Guo et al. 2017a), to generate a strain growing on cellulose. But before reaching the consensus strain, multiple optimization studies were conducted. It was for example observed that CBHI from *T. reesei* was not satisfactorily produced, so an alternative was chosen from among PfCBHI and NcCBHI (Pf—*Penicillium funiculosum*). By playing with the promoter strength (pTEF or hp4d), the relative proportion of the extracellular activity was tailored to optimally hydrolyze cellulose pulp (Organosolv). It was observed that the use of a hybrid promoter instead of the initially used TEF promoter procured four and eight times higher expression of NcCBHI and TrCBHII, respectively. While basic expression level from pTEF was sufficient to ensure recombinant’s growth on CMC cellulose, only strains optimized in expression level of TrCBHII and NcCBHI grew satisfactorily on Avicel and PASC. Nevertheless, the authors concluded that the cellulase combinations used in that study were insufficient for decomposition of crystalline cellulose. That, most probably, inspired the following study, where, in order to improve the conversion yield of recalcitrant cellulose, the authors overexpressed accessory proteins, identified previously as enhancing efficiency of cellulose hydrolysis (Guo et al. 2017b). The following three activities were chosen: (1) lytic polysaccharide monooxygenase (LPMO from *T. reesei*) that catalyzes oxidative cleavage of insoluble polysaccharides; (2) xylanase (XYNII from *T. reesei*) breaking down hemicellulose in lignocellulosic biomass, uncovering cellulose microfibrils; (3) swollenin (SWO1 from *T. reesei*) which is a non-enzymatic protein that disrupts organized structure of crystalline cellulose. In addition, the ratio of the core cellulases cloned previously was further optimized by placing all the cellulases under the control of the strong hybrid promoter (HTEF) except for EGII. Consequently, it was observed that the expression of TrLPMOA greatly enhanced hydrolysis of recalcitrant cellulose substrates, in contrast to TrSWO1, which did not bring any measurable improvement. The resultant strain, with optimized expression of the core cellulases (EGI, EGII, CBHI, CBHII, BGL1, BGL2) and expression of TrXYNII and TrLPMOA, was able to degrade an array of different cellulosic substrates faster than commercial cellulolytic cocktail. In the last work out of this series, stability of the heavily modified strain was improved by changing the mode of the subsequent genes cloning (Guo et al. 2018) as the previously generated strain tends to be unstable and the heterologous gene losses were observed. Along with improved stability, further optimization of the core cellulase expression was conducted by, for example, enhancing expression of BGLs. While the maximum specific growth rates were impaired by overexpression of the six required genes, the cellulose-degrading strains were engineered for production of three high-value products, lipase, lipids, and ricinoleic acid, and successfully used in actual CBP processes with cellulosic substrate (Guo et al. 2018).

### Xylan

Xylan is the most abundant hemicellulose in lignocellulosic biomass, from among the other hemicelluloses, including glucuronoxylan, arabinoxylan, glucomannan, and xyloglucan. Xylan’s backbone is composed of β-(1,4)-linked d-xylose residues, and can be branched through β-(1,3)- or β-(1,3, 1,4)-glycosidic bonds with d-xylose residues or the other carbohydrates, like d-arabinose, d-galactose, or acidified forms (glucuronic acid and galacturonic acid). Xylans are highly abundant in all types of lignocellulosic biomass, including wood, grasses, cereals, and herbs, making up to 35% of its dry weight content (Binod et al. 2011; da Silva et al. 2012). As such, it would be highly desired to use it as a bioconversion substrate in biotechnology, which would significantly increase the economics of biomass utilization. Marketed xylan is mainly obtained from straw, sorghum, sugarcane, corn stalks and cobs, and hulls and husks from starch production, as well as pulping waste products from hardwoods and softwoods (da Silva et al. 2012). Xylan gained attention as a substrate in production of packaging films impenetrable to oxygen, food coatings and emulsifier, in biomedical products for microencapsulation, as well as a component of adhesives, thickeners, and as a plastic additive (da Silva et al. 2012). Due to its abundance, low price, and no competition with the food sector, hemicellulosic xylan holds promise of sustainable substrate for biotechnological conversions. Currently, there is no self-sufficient process or technology available to process the lignocellulosic xylan into value-added products and its hydrolysis into utilizable sugars is the key stage that determines the overall process efficiency (Binod et al. 2011). On the other hand, presence of hemicellulose in lignocellulosic biomass impedes accessibility of cellulose to enzymatic hydrolysis, hence, removal of xylan would be beneficial for cellulose bioconversions.

**Fig. 4:**
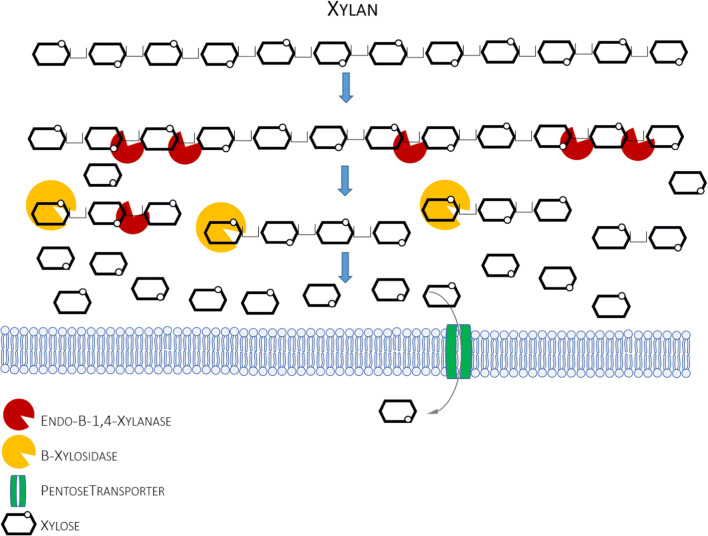
Simplified scheme of enzymatic decomposition of xylan. Only those enzymatic activities that were engineered in *Y. lipolytica* (or the elements studied in *Y. lipolytica*, like protein transporters) are depicted in this simplified scheme

Due to its heterogeneity and branched structure, enzymatic degradation of xylan requires multiple different enzymatic activities acting synergistically. For degradation of homopolymer chains (Fig. 4), endo-1,4-β-xylanase, which cleaves internal glycosidic bonds releasing oligomers, and β-xylosidase, acting on the non-reducing terminus of oligosaccharides, are necessary. Furthermore, α-arabinofuranosidase, α-glucuronidase, and acetyl xylan esterase are needed to degrade the heteropolymer. Commercial xylanase cocktails are mainly obtained from *A. niger, T. reesei, Bacillus* spp., and *H. insolens*. No native ability to degrade xylan has been reported for *Y. lipolytica* strains (Duquesne et al. 2014). On the other hand, the genome mining and the following experimentation revealed presence of a dormant pathway for xylose (monomer of xylan) utilization in *Y. lipolytica*’s genome (Ledesma-Amaro et al. 2016; Ryu et al. 2016; Ryu and Trinh 2018). Native xylose utilization genes in *Y. lipolytica* comprise xylose reductase (XYR/XYL1; YALI0D07634g), xylose dehydrogenase (XDH/XYL2; YALI0E12463g), xylulokinase (XKS/XYL3; YALI0F10923g), and a panel of 16 putative xylose transporters which were shown to be highly induced in the presence of xylose (YALI0B21230g, YALI0F19184g, YALI0A01958g, YALI0A08998g, YALI0F23903g, YALI0C08943g, YALI0F18084g, YALI0B06391g, YALI0D00132g, YALI0F06776g, YALI0B17138g, YALI0F25553g, YALI0D01111g, YALI0C04730g, YALI0D00363g, YALI0B00396g) (Ryu et al. 2016)*.* Noteworthy, a high level of synergistic cross-activation was observed between identified putative transporters for glucose, xylose, cellobiose, and arabinose (Ryu et al. 2016; Ryu and Trinh 2018). Of high relevance to xylan assimilation is an observation that *Y. lipolytica* can simultaneously utilize arabinose and xylose, and that the two sugars share transporters and the other elements of the degradation pathway, like xylitol/arabitol dehydrogenase (YALI0E12463g) (Ryu and Trinh 2018). For details on C5 monosugar utilization by *Y. lipolytica*, the reader is referred to a recent review paper on this subject (Spagnuolo et al. 2018) and highly informative original papers (Ryu et al. 2016; Ryu and Trinh 2018).

As in the case of the abovementioned polymeric substrates, engineering of a xylan-utilization trait in *Y. lipolytica* was in fact initiated by using xylanases as enzymatic reporters. With this aim, xylanase I from *H. insolens* (Muller et al. 1998), XlnC from *A. niger* (Dulermo et al. 2017), or *XYN* from bacterium *Thermobacillus xylanilyticus* (Duquesne et al. 2014) were cloned in *Y. lipolytica*. In the latter study, the protein was expressed under oleic acid–induced promoter and anchored on the cell surface using three different docking domains (CWP, Pir, CBM). Of key importance was the experiment on compromising temperature and pH settings between the optima for the enzyme and the yeast. Reaching consensus in this matter precludes exploitation of a recombinant cell as the consolidated biocatalyst. For the bacterial XYN protein, the optimal was pH 6.0 and the temperature of 60 °C, which is not feasible for *Y. lipolytica*. That problem was considered also in the second study, where endo- and exo-β-(1,4)-xylosidases were cloned in *Y. lipolytica* (Wang et al. 2014). The key criterion for the heterologous enzymes selection was similarity of growth optima between the source organism and *Y. lipolytica*. Consequently, XynII from *Trichoderma harzianum* and XlnD from *A. niger* were chosen for the endo- and exo-acting xylanase, and were cloned separately. Strikingly, sole expression of XynII in *Y. lipolytica* enabled its growth on mineral medium with birchwood xylan as the sole carbon source. On the other hand, co-culturing of XynII- and XlnD-expressing strains resulted in higher degree of xylan degradation. Noteworthy, since no other genes were cloned and the growth was supported, the native xylose-utilization pathway of *Y. lipolytica* had to be spontaneously activated.

### Inulin

Inulin is a water-soluble d-fructose polymer of polymerization degree from 20 up to several thousand units linked by β-(2,1) glycosidic bonds, typically bearing glucose linked through α-(1,2) at a terminus. In the nature, inulin is produced by different plants as a storage material in roots and tubers. Among an array of different inulin-storing plants, like agave, asparagus, coffee, chicory, dahlia, dandelion, garlic, and onion, Jerusalem artichoke is gaining the biggest interest for biotechnological conversions (Hughes et al. 2017). Inulin is currently mainly used for the production of prebiotic fructooligosaccharides that are in high demand worldwide. Agave juice that is rich in inulin is used to produce tequila. Artichoke inulin is exploited in production of fructose for further applications in the food industry. The processes are all executed with microbial inulinases, usually originating from *Kluyveromyces*, *Penicillium*, *Aspergillus*, *Pseudomonas*, or *Clostridium* (Hughes et al. 2017).

**Fig. 5:**
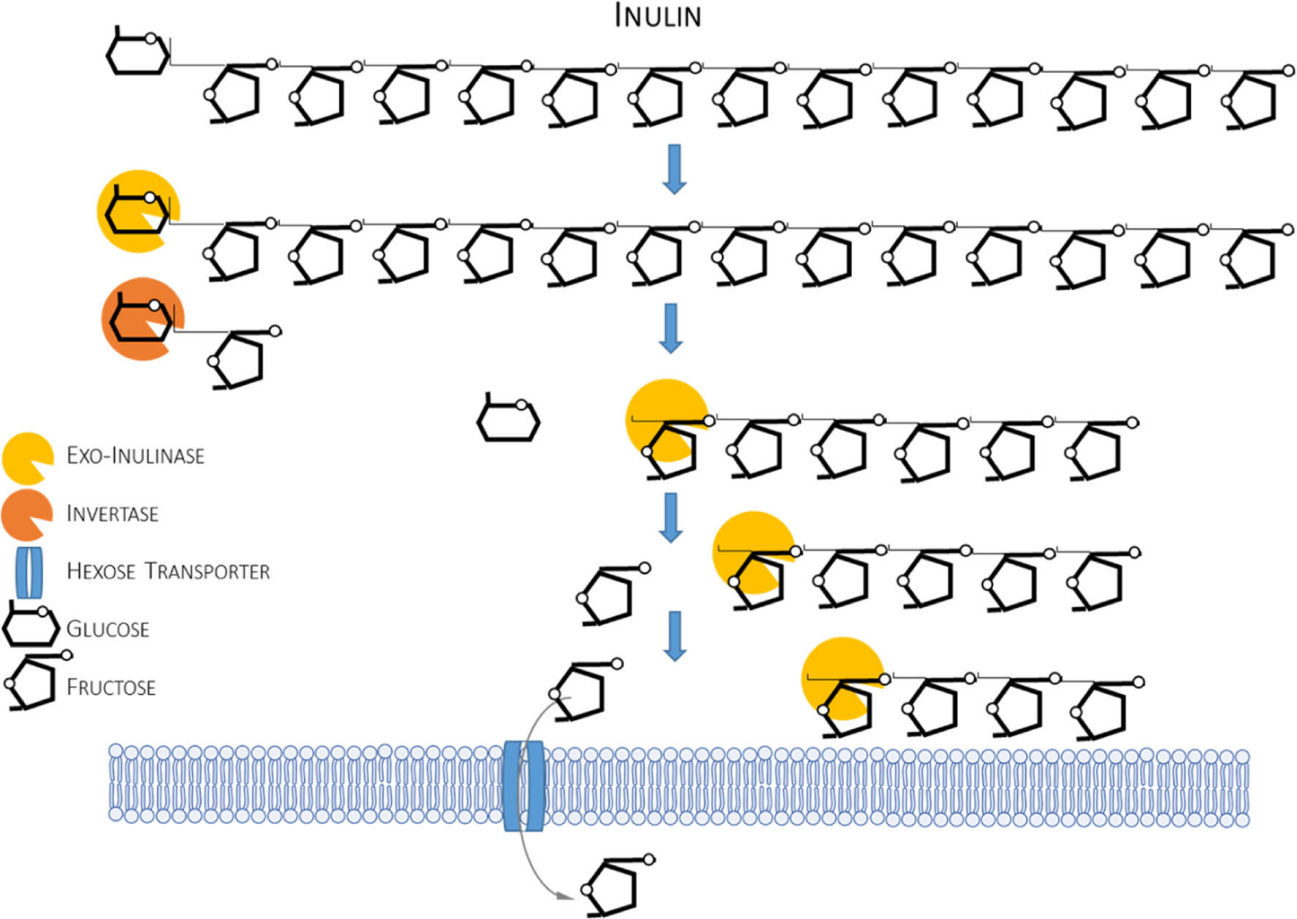
Simplified scheme of enzymatic decomposition of inulin. Only those enzymatic activities that were engineered in *Y. lipolytica* (or the elements studied in *Y. lipolytica*, like protein transporters) are depicted in this simplified scheme.

Two types of enzymatic activities are required to decompose polymeric inulin: endo-acting inulinase yielding fructooligosaccharides and exo-inulinase that hydrolyzes terminal bonds at a non-reducing end and releases monomeric fructose (Fig. 5). Of high relevance to this review is that invertases (typically known for their sucrose-hydrolyzing activity) also have an exo-inulinase activity and hydrolyze fructooligosaccharides of a low degree of polymerization. Direct impact of *S. cerevisiae*’s SUC2 invertase on inulin hydrolysis rate was clearly evidenced (Yang et al. 2015). No inulinase activity has been identified in *Y. lipolytica* (Kurtzman and Fell 2006). Interestingly, *Y. lipolytica* shows high inter-strain variation in terms of growth on fructose (inulin’s basic building block), which is attributed to variation in hexokinase activity (Lazar et al. 2014; Lazar et al. 2017). As revealed recently by genome mining and experimentation, *Y. lipolytica* bears six hexose transporters, with two having clearly defined role in fructose transportation (YHT1/YHT4) (Lazar et al. 2017).

Although this review primarily concerns engineering efforts toward endowing *Y. lipolytica* with polysaccharide-degrading ability, it would suffer from lack of comprehensiveness if cloning of invertase *SUC2* would not be briefly mentioned, due to the enzyme’s intrinsic exo-inulinase activity mentioned above. Cloning of *S. cerevisiae*’s *SUC2* was initially executed with the aim to use an acquired sucrose-hydrolytic trait as a dominant marker (Nicaud et al. 1989) or the enzymatic activity as a reporter (Hong et al. 2012), to grow the recombinant strain on sucrose (Förster et al. 2007) or in sucrose-rich waste stream—molasses (Lazar et al. 2011; Lazar et al. 2013; Gajdoš et al. 2015; Rakicka et al. 2017). No information on testing the *SUC2*-overproducing *Y. lipolytica* strains toward inulin were reported there. This issue was however raised in the following study (Han et al. 2017) showing that this particular invertase (SUC2) produced by *Y. lipolytica* shows only marginal specificity toward inulin and fructooligosaccharides. It was determined that the hydrolytic activity of the SUC2 invertase toward fructooligosaccharides was only 8.8% of that toward sucrose, and there was no detectable hydrolytic activity toward inulin (Han et al. 2017).

The first report on cloning of an inulinase gene (*INU1* from *Kluyveromyces marxianus*) in *Y. lipolytica* was conducted in an environmental isolate strain (mutated to a *ura-* form), known to be efficient in the production of citric acid (Liu et al. 2010). Precise definition of the background strain is highly relevant here, due to the inter-strain variation in fructose assimilation capacity reported previously (Lazar et al. 2014, 2017). The obtained recombinant strain, displaying the INU1 enzyme on the cell surface, could efficiently grow on inulin from Jerusalem artichoke, consume the substrate at high rates, and produce substantial amounts of citric acid (Liu et al. 2010) and lipids (Zhao et al. 2010). The same strain was further modified to optimize production of citric acid from inulin; however, the modifications were related to TCA cycle and to the substrate utilization improvement (Liu et al. 2013). The *K. marxianus*’ gene *INU1* was also cloned in another background—the high citric acid producer AWG7 strain, isolated from a Polish lineage strain *Y. lipolytica* A-101-1.31 after its exposure to UV irradiation (Rakicka et al. 2016). The inulinase enzyme was immobilized on the cell surface as well. The obtained inulinase-hydrolyzing strain was first used for the production of erythritol and citric acid in inulin/glycerol co-substrate medium, resulting in high amounts of the target products (Rakicka et al. 2016). In the following study, seven *INU1*-bearing *Y. lipolytica* sub-clones, presumably differing in genomic integration site of the *INU1*-expression cassette, were compared in terms of inulin consumption rate (Rakicka et al. 2019). While the recombinant (and the parental) strains did not differ in terms of fructose utilization, which is of high importance for inulin utilization tests, they differed significantly in terms of inulin degradation capacity. The best inulin consumer was forwarded to bioprocess intensification studies finally yielding over 200 g/L of citric acid from inulin (Rakicka et al. 2019). *Y. lipolytica*’s high capacity to synthesize secretory protein was used to overproduce inulinases excreted to the culture medium with the aim to obtain inulinolytic enzymatic preparation. Initially, the genes encoding exo-inulinase from *K. marxianus* and endo-inulinase from *A. niger* were cloned separately in a laboratory Po1h strain (Liu et al. 2016). Obviously, solely for overproduction of enzymatic protein, the strain’s background and the associated fructose assimilation capacity lacks importance, as the two proteins were purified from the supernatant. The two proteins were overproduced separately and their activities were analyzed independently and in combination, demonstrating high synergy in their action (Liu et al. 2016). The same expression host was used in another study, where two copies of *A. niger* endo-inulinase were cloned and overexpressed under strong growth-phase-dependent promoter (Han et al. 2017). However, in this approach, the host system was actually used in situ for inulin decomposition, which was executed by a two-stage approach. The first stage was oriented toward overproduction of the enzyme until the culture reached stationary phase, and subsequently, industrial substrate rich in inulin was added to the culture. During this stage, the heterologous endo-inulinase activity hydrolyzed the material to fructooligosaccharides, which were the target product. Notably, in the latter stage, the temperature was raised from initial 28 to 35 °C, which is more suited for the enzyme’s action. Consequently, the authors developed a highly efficient process of fructooligosaccharide production, using recombinant *Y. lipolytica* strain overproducing two fungal endo-inulinases, and SU2 invertase (Po1h background), which removed any non-prebiotic saccharides generated during the process (Han et al. 2017). The abovementioned previous studies on inulinolytic *Y. lipolytica* (Zhao et al. 2010; Liu et al. 2016) were finalized by a work on construction of a strain overproducing both exo- and endo-inulinase simultaneously, able to accumulate 48.13% (g/gDCW) from inulin (Shi et al. 2018). The double recombinant was compared in terms of exerted inulinase activity with the previously constructed strain (exo-inulinase *INU1* solely) and was shown to bear over 2.5-fold higher hydrolytic activity toward inulin than the latter, which well corresponds with the finding on synergistic activity of exo- and endo-inulinases (Liu et al. 2016).

## Summary and outlook

Over decades of intensive worldwide effort, *Y. lipolytica* has been turned into a versatile consolidated biocatalyst, with numerous areas of applications. The discussed above studies aimed at expanding native substrate range of the species to highly abundant polysaccharides, i.e., starch, cellulose, xylan, and inulin. Due to the review capacity, at least several highly promising issues had to be neglected, like acquired capacity to decompose pectins by overexpression of polygalacturonase (Muller et al. 1998), or glucomannan, through surface display of mannosidase (Moon et al. 2013). Based on conducted literature search and own experience, the key challenge in developing *Y. lipolytica*–based consolidated biocatalysts is balancing optimal conditions for the host growth and the enzyme activity. The bioprocesses are typically conducted under conditions facilitating the host growth, neglecting the enzymatic catalyst requirements. As demonstrated in the series of works on cellulolytic *Y. lipolytica* by the French group, consideration of accessory enzymatic and non-enzymatic proteins may importantly advance the generation of a truly efficient consolidated biocatalyst. Likewise, optimization of multi-hydrolyses-producing strains, in terms of a respective activity abundance, seems to be the key point, enabling generation of strains tailored for a specific substrate.

## Supplementary Information

ESM 1 (PDF 394 kb)
