# Supplementary material for: Hydrolytic secretome engineering in Yarrowia lipolytica for consolidated bioprocessing on polysaccharide resources: review on starch, cellulose, xylan, and inulin
Source: Appl Microbiol Biotechnol. 2021 Jan 15;105(3):975–89. doi: 10.1007/s00253-021-11097-1 (PMC7843476; doi:10.1007/s00253-021-11097-1)
Supplement: Supplementary file 1 — (PDF 394 kb) [file 253_2021_11097_MOESM1_ESM.pdf]

Online Resource

Applied Microbiology and Biotechnology

Hydrolytic secretome engineering in *Yarrowia lipolytica* for consolidated bioprocessing on polysaccharide resources: review on starch, cellulose, xylan and inulin

Ewelina Celińska[1*], Jean-Marc Nicaud[2], Wojciech Białas[1]

[1]Department of Biotechnology and Food Microbiology, Poznan University of Life Sciences, ul. Wojska Polskiego 48, 60-627 Poznań, Poland

[2]Micalis Institute, INRA-AgroParisTech, UMR1319, Team BIMLip: Integrative Metabolism of Microbial Lipids, Domaine de Vilvert, 78352 Jouy-en-Josas, France

*Corresponding author: Ewelina Celińska, phone: +48618466007, e-mail: ewelina.celinska@up.poznan.pl, ORCID 0000-0001-8372-8459

Electronic Supplementary Material_1

Comparison of Material Consumption and Material Cost for model processes conducted according to the CBP concept with a recombinant *Y. lipolytica* (1) and enzymatic cocktail based approach with native *Y. lipolytica* (2).

1. Material Consumption

1.1. CBP process (410 batches /yr)

Material	MT/yr	MT/batch	MT/kg MP
Air	6,448	15.727909	0.031456
Amm.chloride	6	0.014430	0.000029
H2SO4 (10% w/w)	1,709	4.167141	0.008334
Inulin	296	0.721525	0.001443
KH2PO4	1	0.001804	0.000004
Lime (33%)	405	0.987733	0.001975
Magne Sulfate	3	0.007215	0.000014
NaOH (1 M)	606	1.478762	0.002958
Nitrogen	44	0.106586	0.000213
Water	4,540	11.073462	0.022147
YE	3	0.007215	0.000014
TOTAL	14,060	34.293783	0.068588

Table S1. Material Consumption in CBP process

1.2 Enzymatic cocktail-based process (148 batches / yr)

Material	MT/yr	MT/batch	MT/kg MP
Air	2,326.433595	15.719146	0.034931
Amm.chloride	2.135714	0.014430	0.000032
H2SO4 (10% w/w)	555.063208	3.750427	0.008334
Inulin	106.785693	0.721525	0.001603
Inulinase	0.267395	0.001807	0.000004
KH2PO4	0.266964	0.001804	0.000004
Lime (33%)	131.566024	0.888960	0.001975
Magne Sulfate	1.067857	0.007215	0.000016
NaOH (1 M)	218.913460	1.479145	0.003287
Nitrogen	15.774679	0.106586	0.000237
Water	1,614.570585	10.909261	0.024243
YE	1.067857	0.007215	0.000016
TOTAL	4,973.913031	33.607520	0.074683

Table S2. Material Consumption in Enzymatic cocktail-based process

2. Material Cost

2.1. CBP process (410 batches /yr)

Bulk Material	Unit Cost ($)	Annual Amount		Annual Cost ($)	Cost ($/kg MP)	%
Air	0.011	5,000,937	m3(STP)	55,010	0.27	4.52
Amm.chloride	0.590	5,830	kg	3,440	0.02	0.28
H2SO4 (10% w/w)	0.014	1,683,525	kg	23,233	0.12	1.91
Inulin	3.570	291,496	kg	1,040,641	5.15	85.46
KH2PO4	3.180	729	kg	2,317	0.01	0.19
Lime (33%)	0.028	399,044	kg	11,069	0.05	0.91
Magne Sulfate	2.100	2,915	kg	6,121	0.03	0.50
NaOH (1 M)	0.084	597,420	kg	50,243	0.25	4.13
Nitrogen	0.000	43,061	kg	0	0.00	0.00
Water	2.000	4,474	MT	8,947	0.04	0.73
YE	4.280	2,915	kg	12,476	0.06	1.02
TOTAL	-	-	-	1,213,498	6.01	100.00

Table S3. Material Cost in CBP process

2.2 Enzymatic cocktail-based process (148 batches / yr)

Bulk Material	Unit Cost ($)	Annual Amount		Annual Cost ($)	Cost ($/kg MP)	%
Air	0.011	1,831,006	m3(STP)	20,141	0.30	3.56
Amm.chloride	0.590	2,136	kg	1,260	0.02	0.22
H2SO4 (10% w/w)	0.014	555,063	kg	7,660	0.12	1.36
Inulin	3.570	106,786	kg	381,225	5.72	67.46
Inulinase	450.000	267	kg	120,328	1.81	21.29
KH2PO4	3.180	267	kg	849	0.01	0.15
Lime (33%)	0.028	131,566	kg	3,650	0.05	0.65
Magne Sulfate	2.100	1,068	kg	2,242	0.03	0.40
NaOH (1 M)	0.084	218,913	kg	18,410	0.28	3.26
Nitrogen	0.000	15,775	kg	0	0.00	0.00
Water	2.000	1,615	MT	3,229	0.05	0.57
YE	4.280	1,068	kg	4,570	0.07	0.81
TOTAL	-	-	-	563,565	8.46	100.00

Table S4. Material Consumption in Enzymatic cocktail-based process
